# Thermal storage properties of lightweight concrete incorporating phase change materials with different fusion points in hybrid form for high temperature applications

**DOI:** 10.1016/j.heliyon.2020.e04863

**Published:** 2020-09-06

**Authors:** Piti Sukontasukkul, Teerawat Sangpet, Moray Newlands, Doo-Yeol Yoo, Weerachart Tangchirapat, Suchart Limkatanyu, Prinya Chindaprasirt

**Affiliations:** a Construction and Building Materials Research Center, Department of Civil Engineering, King Mongkut's University of Technology North Bangkok, Bangkok, Thailand; b Department of Mechanical and Aerospace Engineering, King Mongkut's University of Technology North Bangkok, Bangkok, Thailand; c School of Science and Engineering, University of Dundee, UK; d Department of Architectural Engineering, Hanyang University, Seoul, South Korea; e Department of Civil Engineering, Faculty of Engineering, King Mongkut's University of Technology Thonburi, Thailand; f Department of Civil Engineering, Prince of Songkla University, Hat Yai, Songkhla Thailand; g Sustainable Infrastructure Research and Development Center, Department of Civil Engineering, Faculty of Engineering, Khon Kaen University and Thailand and Academy of Science, The Royal Society of Thailand, Dusit, Bangkok, Thailand

**Keywords:** Civil engineering, Materials science, Construction engineering, Concrete technology, Materials property, Physical property, Thermal storage, Phase change material, PCM in Hybrid form, Thermal conductivity, Latent heat

## Abstract

In this study, the thermal storage properties of lightweight concrete incorporating two types of phase change materials (PCM) with two different fusion points were investigated. Two types of PCM, polyethylene glycol (PEG) and paraffin (PRF), were impregnated into porous aggregates using high temperatures. The PCM aggregates were mixed with concrete at different proportions of PEG/PRF aggregates from 0/100 to 100/0 with 25% intervals. The experimental series consisted of thermal property tests (such as thermal conductivity, specific heat, and latent heat), and some basic properties (such as compressive strength, density, water absorption, and abrasion resistance). The results showed that incorporating PCM aggregates into lightweight concrete helped increase the workability, lower the moisture absorption, and increase the mechanical properties. For thermal properties, both thermal conductivity (k) and specific heat were found to depend strongly on the state of PCM. The latent heat of lightweight concrete with PCM aggregates in hybrid form were found to be higher than that of single type PCM aggregates.

## 1 Introduction

For some engineering applications such as curing room for precast concrete components or concrete blocks, concrete structures may be required to store large amounts of heat inside at a high temperature for long periods of time. In order to do that, such structures are usually constructed with thick, massive walls to extend the duration of heat transfer and maintain a stable high temperature inside. However, this structural form is often not economical because increased mass comes with additional self-weight loadings on foundations and footings.

To enhance insulation properties of walls without compromising the thickness, materials with high energy storage such as phase change materials (PCM) can be used. PCMs are materials capable of storing heat during their phase changing transition. In the process of heating up, the PCM's phase changes from solid to liquid and heat is stored in form of latent heat. Conversely, during the process of cooling down, liquid PCM reverses back to a solid phase and the latent heat is released back to the surrounding environment.

PCMs can be divided into 3 groups: organic, inorganic, and eutectic (combination between organic and inorganic). They are available in a range of fusion temperatures and with a varying latent heat storage capabilities and the selection depends on application requirements. For household use in countries in cold regions, a fusion temperature of 20–25 °C is more suitable. For some applications such precast concrete or aerated concrete block manufacturing, in which the curing process requires high temperatures up to about 60–70 °C for a certain period of time, a higher fusion point PCM will be more desirable.

PCMs have been used as a heat storage medium in several construction materials such as gypsum boards, masonry blocks, plastering mortar or concrete, etc. For example, Hawes et al. [1] investigated properties of PCM in both gypsum boards and masonry blocks. The PCM used in the manufacturing of gypsum boards was directly mixed with gypsum in liquid form by immersion at 80 °C for several minutes. They found that PCM gypsum boards exhibited higher density, lower moisture absorption (by about 33%), and higher energy storage (by around 11 times) compared to conventional boards. For concrete blocks, they used PCM in both powder and liquid forms (immersion). Similar results to the gypsum boards were observed with moisture absorption reducing and energy storage increased by 200–230%.

Salyer and Sircar [2] inserted PCM in dry form into the hollow core space of concrete blocks. This allowed them to experiment with the energy storage of PCM in large quantities. Thermal storage of over 1,000,000 BTU was obtained in a building with dimensions of 10 × 12 × 3 m constructed with PCM-hollow core concrete blocks. They also investigated the effect of PCM on thermal storage of plaster boards [3]. Some other notable research on plaster board incorporating micro encapsulated PCM can be found in references [4, 5] and [6].

In the case of plastering mortar, Sá et al. [7] investigated thermal enhancement of plastering mortar incorporating phase change materials. The PCM was prepared in the form of micro encapsulated spheres and mixed with plastering mortar. Their thermal enhancement was achieved at a PCM content of 25% by mass fraction which yielded enthalpy of 25 kJ/kg in a melting range from 23 °C to 25 °C, and a thermal conductivity of 0.3 W/m◦C. Sukontasukkul et al. [8] investigated the use of PCM with a high fusion point as an insulating agent for use in hot and humid countries like Thailand (where the average temperature is around 28–32 °C). In their experiment, polyethylene glycol with fusion points of around 42–46 °C was mixed with mortar in dry form and used as plastering mortar on exterior walls. They found the delay in time to reach peak temperature of the cubicle plastering with PCM mortar due to the effect of latent by PCM. The delay also caused the peak temperature cubicles plastering with PCM mortar to be slightly lower than those plastering with conventional plastering mortar.

In the case of concrete materials, several methods of incorporating PCM have been proposed. At the beginning, PCM was directly mixed with concrete mixture as a constituent material. However, some drawbacks were observed related to the changes in concrete properties, the interactions between cement paste and PCM, and the PCM leakage [9]. To deal with the leakage problem, the encapsulation technique was introduced [10, 11, 12, 13]. However, because of the poor bond between PCM microcapsules and high porosity, a drop of strength was commonly observed [14].

Our recent studies [15] investigated the use of porous aggregates as PCM carriers instead of microencapsulated spheres. The polyethylene glycol (PEG) with fusion points of around 42–46 °C was impregnated into lightweight aggregates using heat and pressure. This technique allowed PCM to be incorporated in larger volumes of up to about 8.1% of total weight. A latent heat of about 4,800 to 7,200 J/kg was observed depending on the volume of PCM aggregates.

In this study, the thermal properties on lightweight concrete mixed with two types of PCM with two different fusion points was investigated. Since the application of this study aims to develop high insulation lightweight concrete suitable for concrete structures subjected to high temperatures, two types of PCM with high fusion points were used (PEG with fusion point of 42–46 °C and paraffin with fusion point of 56–59 °C). The use of two PCMs allowed the latent heat to be stored at two temperature ranges. The PCM aggregates were prepared by impregnating liquid PCM into porous concrete using high temperatures. Concrete samples were prepared with different proportions between both PCM aggregate types (0:100, 25:75, 50:50, 75:25, and 100:0). The thermal properties such as thermal conductivity, specific heat, and latent heat were determined including some basic properties such as compressive strength, density, water absorption, and abrasion resistance.

## 2 Experimental procedure

### 2.1 Materials

Materials used in this study consisted of Portland cement type I (ASTM C150), fine aggregate passing sieve no.4, and lightweight aggregate with properties given in Table 1. Two types of phase change materials (PCM) with different fusion points were used: Paraffin 6035 (PRF) and Polyethylene glycol 1450 (PEG) with properties as shown in Tables 2 and 3.

**Table 1:** Properties of lightweight aggregates.

Specification	Unit	
Maximum particle size	mm	10
Unit weight	kg/m^3^	732
Percent of voids	%	72
Bulk specific gravity (Dry Basis)		1.08
Bulk specific gravity (SSD Basis)		1.25
Apparent specific gravity		1.3
Apparent porosity	%	18.1
Percent absorption	%	17.5

**Table 2:** Properties of paraffin 6035.

Specification	Unit	
Melting Point	°C	57.2–59.9
Specific Gravity	at 25 °C	0.89
Latent Heat	kJ/kg	189
Thermal Conductivity	W/(m.K)	0.21
Specific Heat	J/kg.K	2100
Oil Content	mass%	0.4
Penetration	at 25 °C	10.0–17.0
Color		30
UV Absorption		1.3

**Table 3:** Properties of Polyethylene Glycol type 1450.

Specification	Unit	
Melting point	°C	42–46
Specific gravity	at 25 °C	1.09
Latent heat	kJ/kg	155
Thermal conductivity	W/m.K	0.23
Specific heat	J/kg.K	2100
Flash point	°C	285
Range of Avg. Molecular Weight		1305–1595
Physical Form		Flake

### 2.2 PCM aggregate preparation

**Figure 1:**
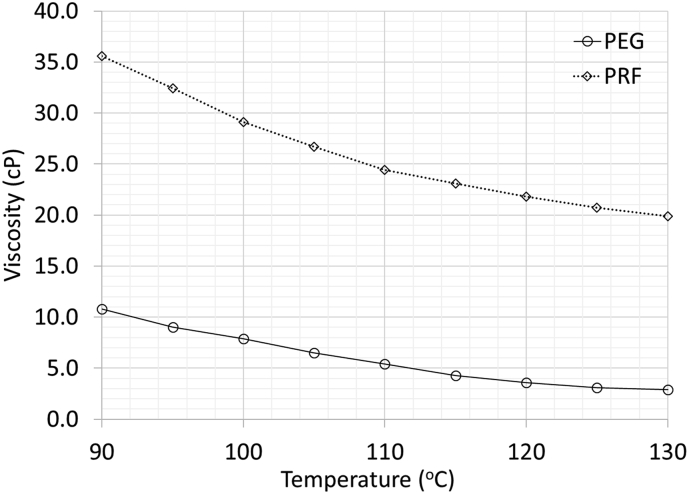
Influence of temperature on viscosity of PCMs used to impregnate aggregates.

The PCM aggregates were prepared by impregnating PCM into aggregates using high temperatures. The process began with heating the PCM (both PRF and PEG) to achieve a liquid state, submerging the lightweight aggregates into the liquid PCM, and then placing them in an oven at a temperature of 120 °C for 8 h. This process created PCM aggregates with impregnation percentages of 17.8 % and 23.5% for PRF and PEG aggregates, respectively. The high temperature caused the viscosity of both PCMs to decrease (Figure 1) allowing the PCM to penetrate inside the aggregates. More details on PCM impregnation process are described elsewhere [15]. The properties of normal and PCM aggregates are compared in Table 4. The PCM aggregates exhibited a large reduction in porosity and an increase in density of around 30% compared to normal lightweight aggregates. The PCM impregnation also enhanced the abrasion resistance of aggregates by about 62–68%.

**Table 4:** Comparison of normal lightweight and PCM Aggregate properties.

Properties	Normal Agg.	PCM Agg.	
		PRF	PEG
Water Absorption (%)	16.0	0.20	0.17
Bulk specific gravity (Dry Basis)	1.068	1.297	1.304
Abrasion Resistance (Percent Weight Loss, %)	31.7	12.1	10.3

### 2.3 Mix proportion

The concrete mix proportion were set at 1:1.02:1.05:0.42 (Cement: Lightweight aggregate: Fine aggregate: Water). For concrete with single type PCM aggregates, the total content of normal lightweight aggregates was replaced with either PRF-PCM or PEG-PCM aggregates. For concrete with two types of PCM aggregates, the PEG-PCM aggregates were replaced with PRF-PCM aggregates in increments of 25% by weight ranging from 0 to 100%. More details are given in Table 5.

**Table 5:** Mix proportions of reference and PCM aggregate concretes.

No.	Type	Weight per Unit Volume (kg/m^3^)					
		Normal LW Agg.	PEG-Agg. (P1)	PRF-Agg. (P2)	Fine Agg.	Cement	Water
1	100L	488	0	0	550	477	200
2	100P_1_/0P_2_	0	595	0	550	477	200
3	75P_1_/25P_2_	0	446	149	550	477	200
4	50P_1_/50P_2_	0	298	298	550	477	200
5	25P_1_/75P_2_	0	149	446	550	477	200
6	0P_1_/100P_2_	0	0	595	550	477	200

Note: L = Normal lightweight aggregate; P_1_ = PEG PCM aggregate; P_2_ = PRF PCM aggregate.

### 2.4 Specimen preparation

The specimens were prepared by dry mixing all ingredients for a few minutes and, after adding water, the mixing continued for further for another few minutes. After mixing finished, the fresh concrete was poured into steel molds in layers, with the number of layers depending on the specimen type. Three specimen types were prepared: cubes with dimensions of 150 × 150 × 150 mm for compression and abrasion tests (EN12390-3), prisms with dimensions of 100 × 100 × 350 mm for flexural test (ASTM C78), and square panels with dimensions of 200 × 200 × 50 for thermal test (ASTM C518 and ASTM C1784). Each layer was compacted with a steel rod for 25 times and vibrated on a shaking table. After completing the top layer, excessive concrete was removed and the specimens were covered with plastic sheets. The specimens were demolded on the next day and wrapped with plastic sheets for 28 days prior to testing.

### 2.5 Experimental series

Each type of concrete panel was subjected to series of test as follow:

• Density test (ASTM C138)
• Water absorption test (ASTM C642)
• Abrasion resistance (ASTM C779)
• Compressive strength (EN12390-3 2002)
• Flexural strength (ASTM C78)
• Steady-state thermal transmission properties by means of heat flow meter apparatus (ASTM C518-17)
• Thermal storage properties of phase change materials and products (ASTM C1784 – 14)

#### 2.5.1 Thermal properties test and data analysis

**Figure 2:**
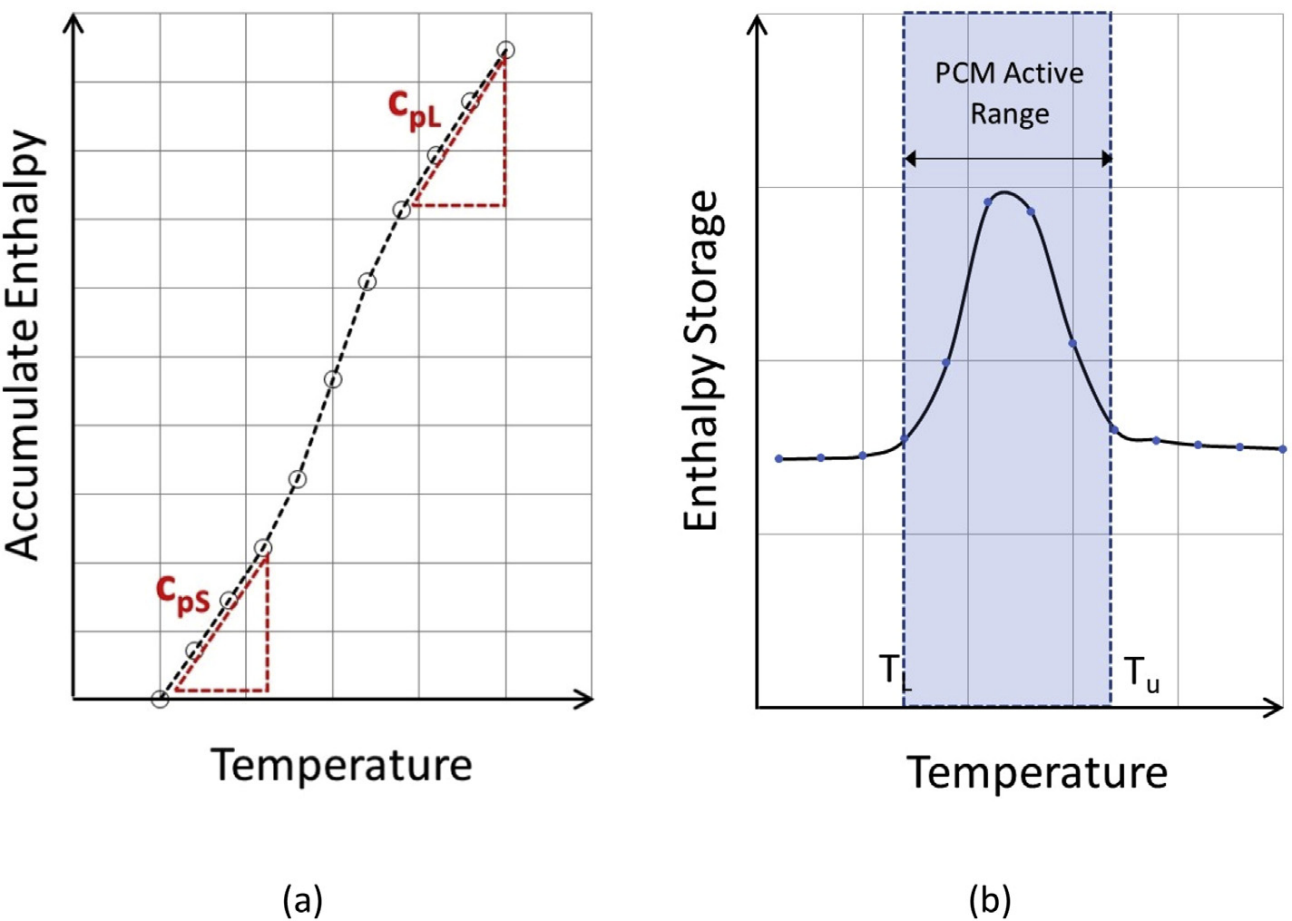
Methodologies for calculating (a) Specific heat and (b) Latent heat.

Two thermal properties (thermal conductivity and latent heat) were investigated using a heat flow meter apparatus constructed at King Mongkut's University of Technology North Bangkok (Figure 2). The apparatus consisted of two hot plates installed with two heat flux sensors and eight K-type thermocouples on both sides. To prevent temperature leakage, an insulating wool with thickness of 50 mm was used in covering around the specimen edges. All sensors and thermocouples were connected to and collected by an automatic data acquisition box.

##### 2.5.1.1 Thermal conductivity

Since the melting temperatures of the two PCMs were different, the two temperature tests were proposed to measure the thermal conductivity of concrete containing PCM aggregates at different stages. The first temperature was selected at 25 °C, which was the temperature low enough to keep both PCM in solid phases. This temperature yielded the thermal conductivity concrete with PCMs in solid stage. On the opposite end, to obtain the thermal conductivity of concrete with all PCMs in liquid stage, a testing temperature of 65 °C was selected which was higher than the melting points of both PCMs.

To begin a test, the two heat plates were set to have equal temperatures. After the heat flux became constant, a new target temperature was set on one of the hot plates. The difference in temperature on both sides allowed the heat to transfer through the sample. The test was stopped as soon as the steady-state was reached. The total heat required for the sample to reach equilibrium (steady-state) at the new target temperature was measured. The thermal conductivity can be calculated by Eq. (1).

(1) $$\lambda_{T}=q.\left(\frac{L}{\text{Δ}T}\right)$$

where λ_T_ is thermal conductivity at any temperature (W/m · K), q is heat flux (W/m^2^), L is the distance between the heat plates (m) and ΔT is temperature difference across the specimen (K).

##### 2.5.1.2 Latent heat

The latent heat can be determined by measuring energy storage in a material over a range of temperatures. The selected range of temperatures must cover the melting range of PCM and divide into steps of 1 or 2 °C. The test began by setting initial temperatures of both hot plates to be identical, and then both heat plates were set to a new identical temperature. The heat flow was measured simultaneously until the temperatures of the hot plates and specimen became identical and the steady-state was achieved. The steady-state is defined as the reduction in the amount of energy entering the specimen from both plates to a very small and near constant value (ASTM C1784). The total energy required to move the temperature up each step was measured and recorded. Similar processes were repeated until the steady state of the final temperature was achieved.

In this study, the temperature range was set from 35 °C to 65 °C with 2 °C incrementing steps to cover the melting temperature of both PRF and PEG. The phase states of each PCM over the entire range of temperature are demonstrated in Table 6 below.

**Table 6:** State of PCMs on temperature range.

Temperature range, °C	Phase State of PCM	
	PEG (P1)	PRF (P2)
35–42	Solid	Solid
42–46	Melting	Solid
46–56	Liquid	Solid
56–59	Liquid	Melting
59–65	Liquid	Liquid

To determine a specific heat, a graph between the cumulative enthalpy storage (CES) and temperature (T) was plotted, then the baseline was drawn by performing a linear regression using the first data and subsequent data points with the regression coefficient (R^2^) smaller than 0.995. The slope of the line connecting the first to the last data point with R^2^ greater than 0.995 was defined as the specific heat values (Figure 2a). In the case of a material containing a single type PCM, two specific heats are commonly determined: C_pS_-specific heat under solid state PCM and C_pL_-specific heat under liquid state PCM. Then latent heat can be calculated using Eqs. (2), (3), (4), (5), and (6).

To obtain the latent heat, the total heat (*H*_*t*_) is defined as being equal to the summation of enthalpy (*ΔH*) over the assigned temperature range (Eq. (2)).

(2) $$H_{t}=\sum_{T_{L}}^{T_{U}}\text{Δ}H$$

In temperature region where the PCMs are not active (i.e. not melting), the total heat or enthalpy stored in the specimen is assumed equal to the sensible heat (*H*_*s*_) (Eq. (3)).

(3) $$H_{t}=H_{s}$$

In the region where a PCM is melting, the latent heat takes affect and the total heat is assumed equal to the combination of sensible (*H*_*s*_) and latent heats (*H*_*l*_) (Figure 2b).

(4) $$H_{t}=H_{s}+H_{l}$$

Over the temperature range where PCM is melting, the sensible heat (*H*_*s*_) can be determined using an average specific heats below and above the active range of the PCM using Eq. (5).

(5) $$H_{s}=\frac{\left(C_{pS}+C_{pL}\right)\left(T_{U}-T_{L}\right)}{2}$$

Based on Eq. (3), The latent heat is the difference between the total heat and the sensible heat as expressed by Eq. (6).

(6) $$H_{l}=H_{t}-H_{s}=\sum_{T_{L}}^{T_{U}}\left(\text{Δ}H\right)-\frac{\left(C_{pS}+C_{pL}\right)\left(T_{U}-T_{L}\right)}{2}$$

where Δ*H* is the enthalpy (J/m^2^), *T*_*U*_ is an upper temperature limit of the PCM active Range (°C), *T*_*L*_ is a lower temperature limit of the PCM active Range (°C).

**Figure 3:**
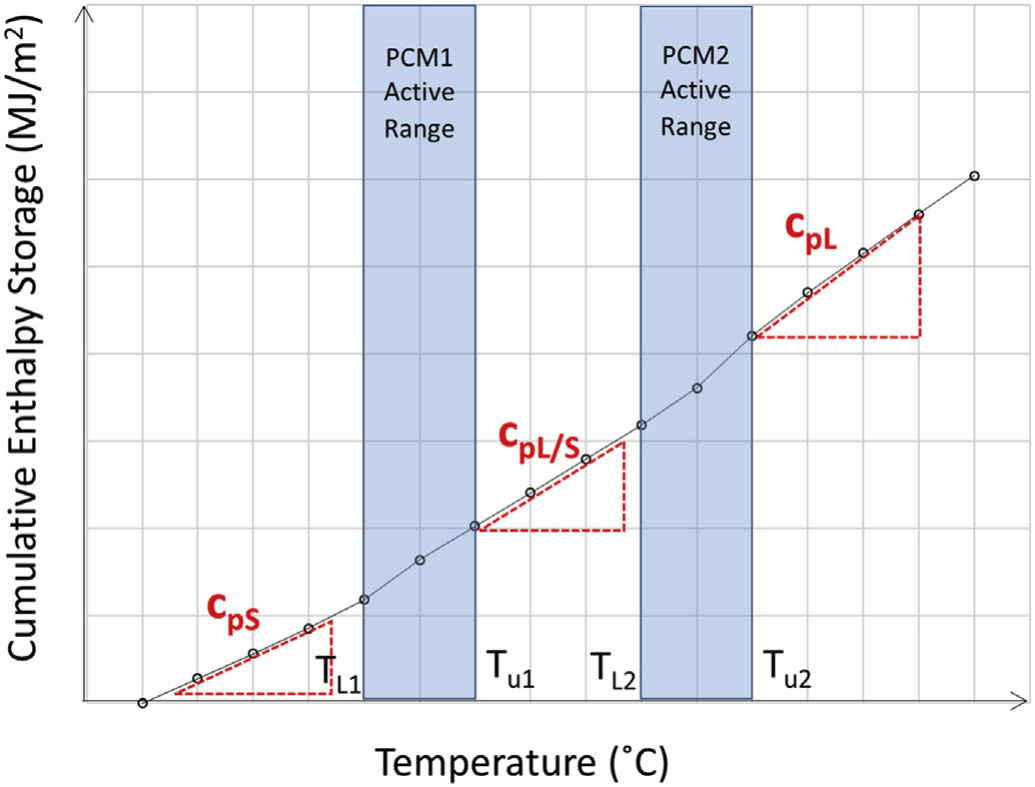
Specific heats at concrete containing PCM in hybrid form.

In the case of concrete containing two types of PCMs, the phase state of PCM is divided into 3 states as shown in Table 6. Based on the phase state of PCM, three specific heats can be determined as C_pS_, C_pL/S_, and C_pL_ as shown in Figure 3., where C_pS_ refers to the specific heat with both PEG and PRF in solid state, C_pL/S_ is the specific heat with PEG in liquid and PRF in solid state, and C_pL_ is the specific heat with both PEG and PRF in liquid state. The latent heat can then be calculated by Eq. (7).

(7) $$H_{l}=H_{t}-H_{s1}-H_{s2}=\sum_{T_{L1}}^{T_{U2}}\left(\text{Δ}H\right)-\frac{\left(C_{pS}+C_{pL/S}\right)\left(T_{U1}-T_{L1}\right)}{2}-\frac{\left(C_{pL/S}+C_{pL}\right)\left(T_{U2}-T_{L2}\right)}{2}$$

## 3 Results and discussion

### 3.1 Workability

**Figure 4:**
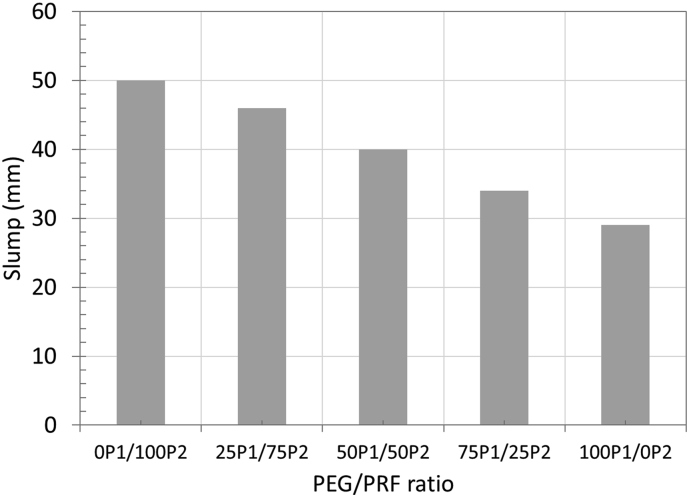
Influence of Replacement of PEG with PRF aggregates on workability.

The workability of concrete was determined by a slump test and the results are given in Figure 4. Considering concrete containing single PCM aggregate type, the concrete with 100%PEG (100P_1_/0P_2_) exhibited larger slump than that with 100%PRF (0P_1_/100P_2_) by about 20 mm. This is partly due to a slightly higher absorption of PRF aggregates (0.20%) as compared to PEG aggregates (0.17%), which allow them to absorb a more water, hence lowered the workability. Another reason could be from the impregnation percentage itself. Since the PEG can be impregnated into aggregates at a higher level than the PRF (by around 5%), this meant that the excessive PCM may have coated the outer surface of PEG aggregates. This coating may have improved the surface smoothness, leading to better particle mobility and higher slump.

In the case of concrete with two PCM types, the slump was directly affected by the variation of PEG and PRF aggregate contents. Since the slump of PEG aggregate was better than that of PRF aggregate, replacing the PEG aggregate with PRF aggregates reduced the slump gradually with increasing PRF aggregate content (Figure 3). The relationship between slump and percentage PRF aggregate content is demonstrated by Eq. (6).

(8) $$S=-0.216\left(\%A_{PRF}\right)+50.6$$

where S is slump (mm), %A_PRF_ is the replacement percentage of PRF aggregate over PEG aggregate (%) ranging from 0 to 100%.

### 3.2 Density and absorption of concretes

**Figure 5:**
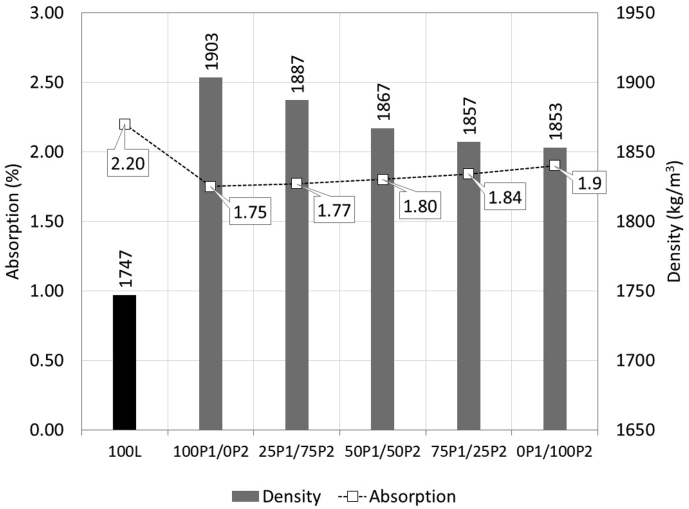
Density and absorption.

Results on the density and absorption of concretes are shown in Figure 5. The density of control lightweight concrete was observed to be 1747 kg/m^3^. The density increased to about 1853–1903 kg/m^3^ when normal aggregates were replaced with PCM aggregates. The densities of PCM aggregate concrete were found to depend on the ratio between PEG/PRF aggregates. The maximum density of 1903 kg/m^3^ was observed in concrete containing 100%PEG aggregate and decreased gradually with the increasing portion of PRF aggregates. The lowest density was observed in concrete containing 100%PRF aggregate at about 1853 kg/m^3^. This is because the specific gravity of PEG aggregates is higher than that of PRF aggregates (Table 4) and by gradually replacing portions of PEG aggregates with PRF aggregates, the density decreased.

In the case of absorption, the normal lightweight concrete (100L) exhibited the highest absorption of 2.20% due to the high porosity and absorption of lightweight aggregates. By replacing the entire amount of normal aggregates with PCM aggregates, the absorption decreased to about 1.75–1.90%. This because PCM aggregates are much denser with less porosity than normal aggregates (Table 4).

Comparing between two types of PCM aggregates, the absorption was found to increase with the increasing PRF aggregate ratio. The mix with 100% PEG aggregates exhibited the lowest absorption of about 1.75%. The absorption then increased gradually with the increasing PRF aggregate content to the highest at about 1.90% in the mix containing 100% PRF aggregate. The increase in absorption was because the absorption of PRF aggregates was higher than that of PEG aggregates by about 17.6% (Table 4).

### 3.3 Abrasion resistance

The abrasion resistance was tested in accordance to ASTM C779. The specimen was subjected to abrasion by pressing dressing wheels, with bearing weight of 7.5 kg, on the horizontal surface of specimen for the period of 30–60 min. The specimen's weight before and after testing were measured and used in determining weight loss percentage over the original weight.

**Figure 6:**
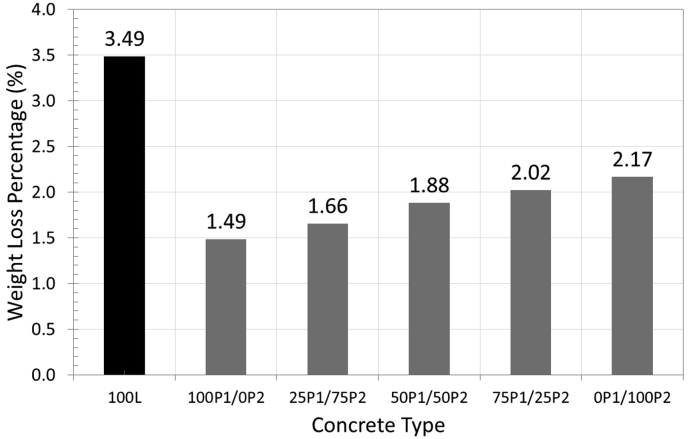
Weight Loss Percentage due to abrasion exposure.

The weight loss percentage of all specimen types were observed to be between 1.5 % and 3.5 % (Figure 6). The concrete with normal lightweight aggregate exhibited maximum weight loss percentage of 2.5%. With PCM aggregates, the weight loss percentage reduced to between 1.5 % and 2.2 % depending on the ratio between P_1_ and P_2_ (Figure 5). Concrete with 100% PEG aggregate (100P1/0P2) exhibited the lowest weight loss percentage of about 1.5%. The weight loss percentage increased gradually with increasing PRF aggregate ratio. The highest weight loss among concrete with PCM aggregates was observed in concrete containing 100 PRF aggregate (0P_1_/100P_2_), the reason being that the abrasion resistance of PRF aggregate was poorer than that of PEG aggregates by about 20% (Table 4) and by replacing PEG aggregate with PRF aggregate, the weight loss percentage increased by a similar proportion.

### 3.4 Compressive strength

**Figure 7:**
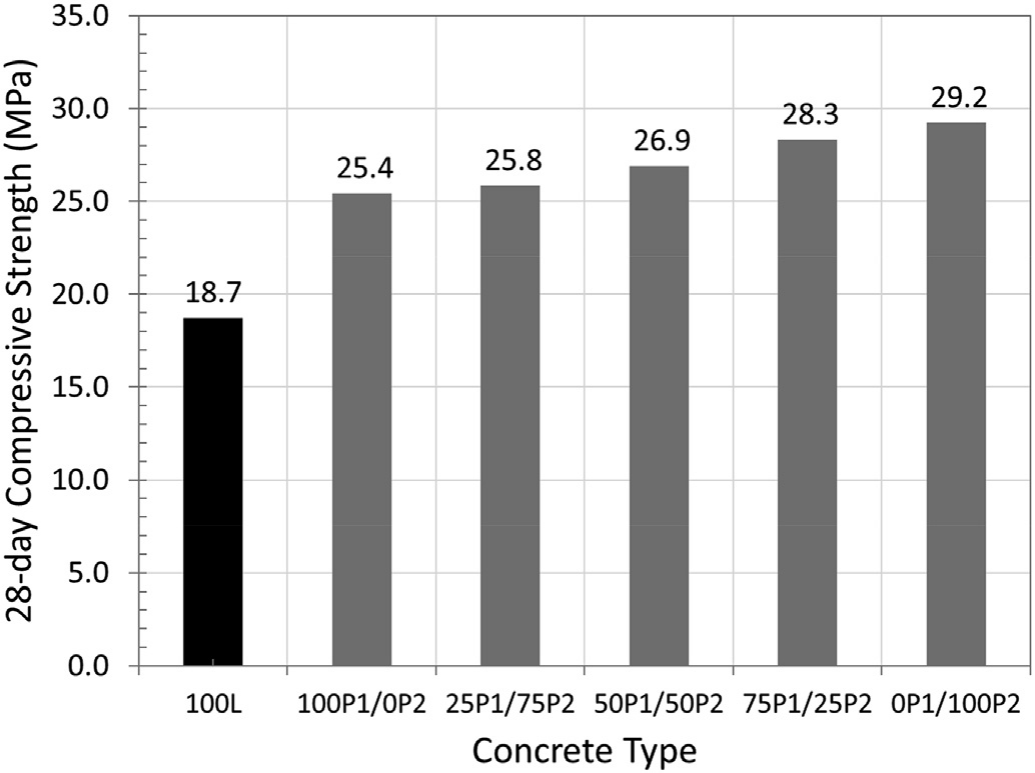
Influence of aggregate type on compressive strength.

The influence of aggregate type on compressive strength is shown in Figure 7. The lowest compressive strength of 18.7 MPa was observed in concrete containing 100% of normal lightweight aggregate (100L). The strength then increased to between 25.4 MPa and 29.2 MPa in concrete containing PCM aggregates. The increase in strength was due to the reduction of porosity in aggregates as they were filled up with PCMs.

The lowest strength amongst the PCM concretes was with 100% PEG aggregates. With the increasing proportion of PRF aggregates, unlike other properties, the compressive strength increased. The strength was highest at 29.2 MPa in concrete containing 100% of PRF aggregates. The lower strength with PEG is believed to be the direct result of the excessive PCM coating at surface of PEG aggregates hence creating a weaker bond between aggregate and cement paste.

### 3.5 Thermal properties

#### 3.5.1 Thermal conductivity coefficient of concretes

**Figure 8:**
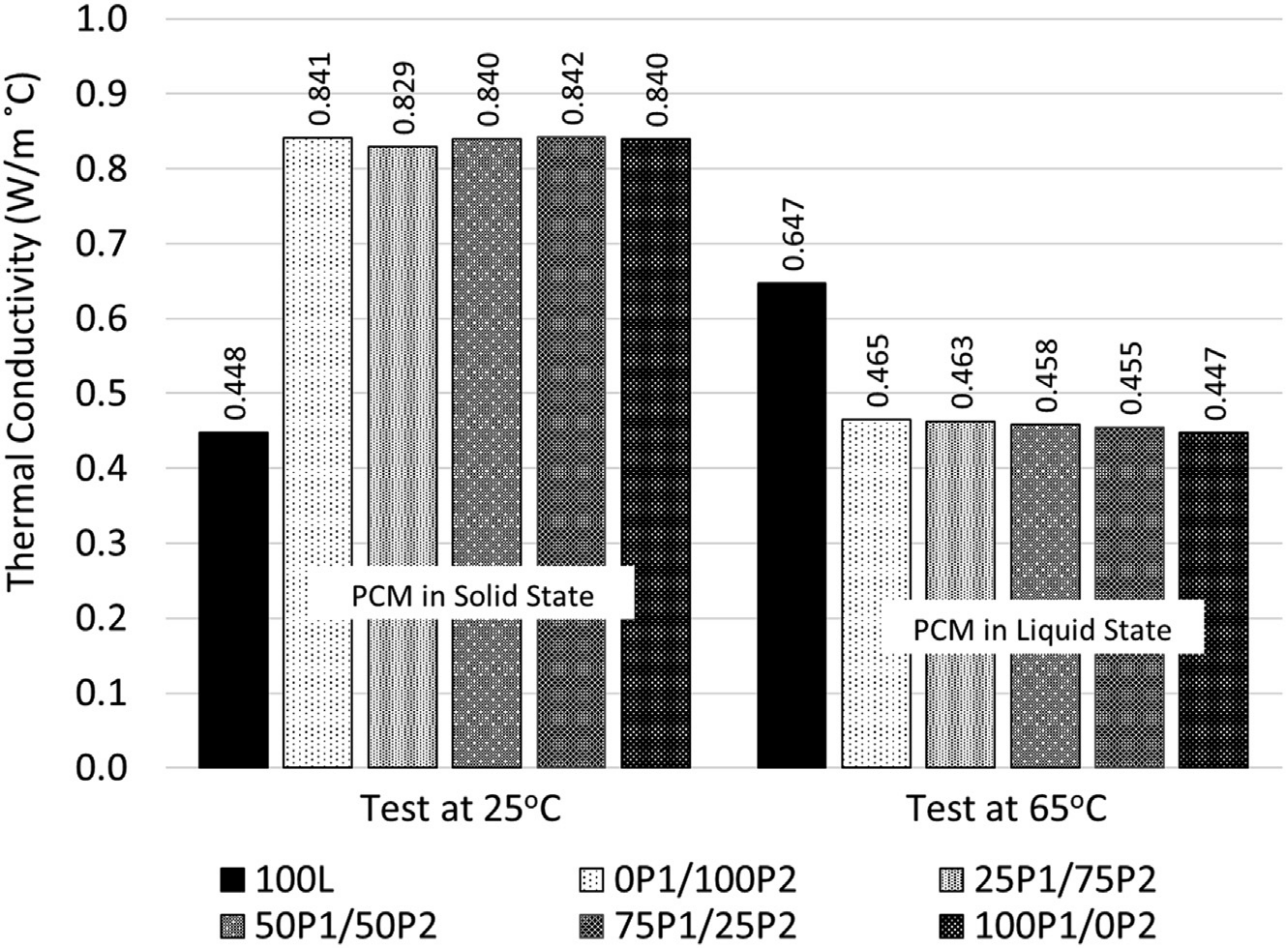
Influence of aggregate type on thermal conductivity.

The thermal conductivity coefficient (k) of concrete was tested under 2 different temperatures in order to study the effect of PCM state (liquid and solid) on k. The 1^st^ temperature of 25 °C was selected because it was lower than the fusion points of both PCMs. This temperature aimed to test for the k of concrete at 25 °C (k_25_) or with PCMs in solid state. The 2^nd^ temperature of 65 °C was selected because it was higher than the fusion points of both PCMs. The temperature, in this case, aimed to measure the thermal conductivity coefficient of concrete at 65 °C (k_65_) or with both PCMs in liquid state. The results of both tests are shown in Figure 8.

For plain lightweight concrete (100L), k_25_ and k_65_ were 0.448 and 0.647 W/m^°^C, respectively. Since 100L contained no PCM aggregates, there no influence of PCM on the k values. The increase in k with the increasing test temperature was the direct result of the temperature itself because the thermal coefficient is known to be sensitive to the testing temperature.

In the case of concrete with PCM aggregates tested at 25 °C, the k_25_ was observed in a similar range between 0.829 - 0.842 W/m^°^C. They appear to be higher than that of 100L by about 40%. The reason should be the effect of PCM in filling up pores inside aggregates. Since, the PCMs are in solid state at this temperature, the filling of PCM reduced the porosity and hence, increased the thermal conductivity of concrete.

For those with PCM aggregates tested at 65 °C, the k_65_ was found in the range of 0.447–0.465 W/m^°^C. This is about 40% lower than the k_65_ of L100. The decrease was the direct effect of PCM phase changing transition. As the temperature increased to 65 °C, both PCMs underwent the phase transition from solid to liquid state, while heat was also stored inside the PCMs during this transition. This stored heat interrupted the normal heat conductivity process and caused the heat to transfer at slower rate and hence, decreased the k_65_ values.

#### 3.5.2 Specific heat of concretes

The specific heat refers to the amount of energy required to raise the temperature of a unit mass of a material up to a certain level (usually 1 °C). For concrete containing one type of PCM, two specific heats can be determined: specific heat of solid PCM (C_pS_) and specific heat of liquid PCM (C_pL_). The C_pS_ refers to the specific heat of a material containing PCM in solid state. It is usually measured prior to the melting temperature of PCM. The C_pL_ is the specific heat measured beyond the melting temperature of PCM which causes the PCM to be in liquid state.

**Figure 9:**
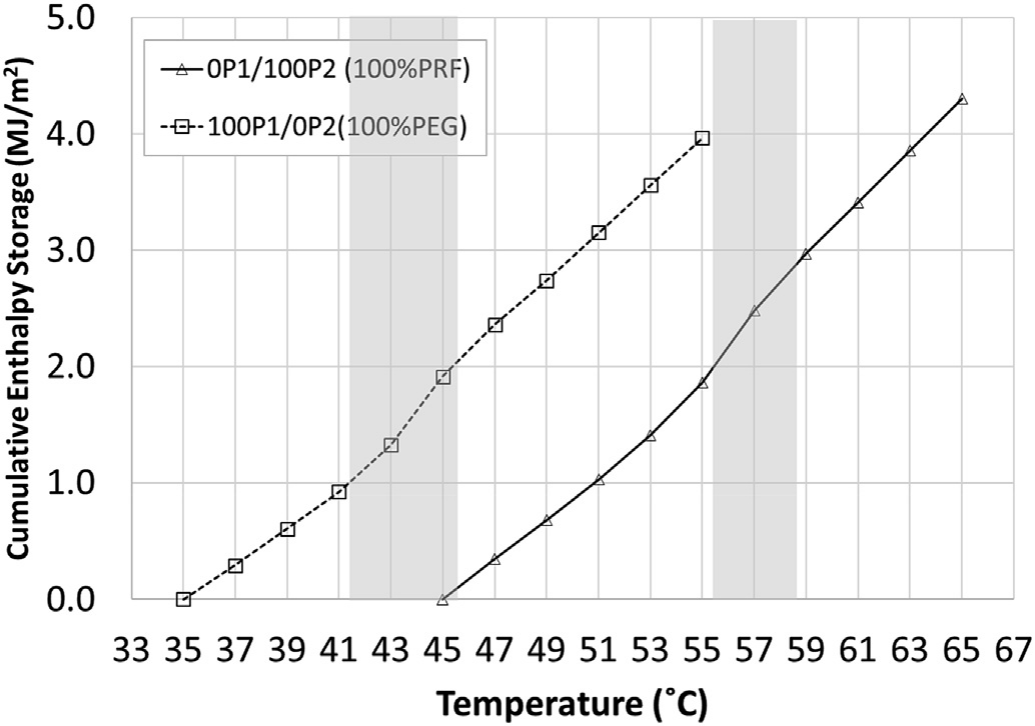
Cumulative Enthalpy and Temperature of Concrete containing Single PCM Type.

Figure 9 shows the relationship between cumulative enthalpy and temperature of concrete containing single PCM type (100P_1_/0P_2_ and 0P_1_/100P_2_). Both concretes exhibited similar behaviours when subjected to heat. At the beginning, a linear relationship between the cumulative enthalpy and temperature was observed. The increase of cumulative enthalpy was linearly proportional to the increasing temperature. At the PCM phase changing transition zone, the change in slope (increase) was observed. This change indicated the interference of PCM to the enthalpy storage, in which higher energy was required to raise the temperature during the PCM phase transition. After all the PCMs changed phase from solid to liquid, the slope dropped back slightly. This indicated the end of PCM influence on the enthalpy storage. Both C_pS_ and C_pL_ obtained from this graph are shown in Table 7.

**Table 7:** Specific Heat of Concrete containing Single Type of PCM.

Concrete Type	PCM System	Specific Heat, (MJ/m^2^)/^o^C		
		C_pS_	C_pL/S_	C_pL_
100P_1_/0P_2_	Single	0.153	-	0.204
75P_1_/25P_2_	Hybrid	0.153	0.198	0.206
50P_1_/50P_2_	Hybrid	0.146	0.195	0.212
25P_1_/75P_2_	Hybrid	0.147	0.187	0.214
0P_1_/100P_2_	Single	0.150	-	0.214

Note:

• C_pS_ is the specific heat where both PCMs are in solid state.

• C_pL/S_ is the specific heat where one PCM is in liquid state and another is still in solid state.

• C_pL_ is the specific heat where both PCMs are in liquid state.

For concrete with single type PCM aggregates, the specific heat was found to depend on the state of PCM and testing temperature. The PCM type appeared to provide no effect of the value of specific heat. Regardless of PCM type and content, both C_pS_ and C_pL_ were found in the range of 0.146–0.153 MJ/m^2^/^o^C and 0.204–0.214 MJ/m^2^/^o^C, respectively. This is because both PEG and PRF possess similar specific heat of about 2100 J/kg.K (Tables 3 and 4).

In terms of PCM state, the C_p_ of both concretes increased as the PCM phase change from solid to liquid. This is strongly related to both the state of PCM and the testing temperature. It is known that the specific heat of a material depends strongly on temperature and state of material. A material tested under liquid state often yields higher C_p_ than the one tested under solid state [16]. In this study, the C_pL_ was found to be higher than the C_pS_ by up to 46% for both types of concrete, also since the testing temperature during PCM liquid state was higher than the one during solid state. The effect of temperature also contributed partly to the increasing of specific heat.

**Figure 10:**
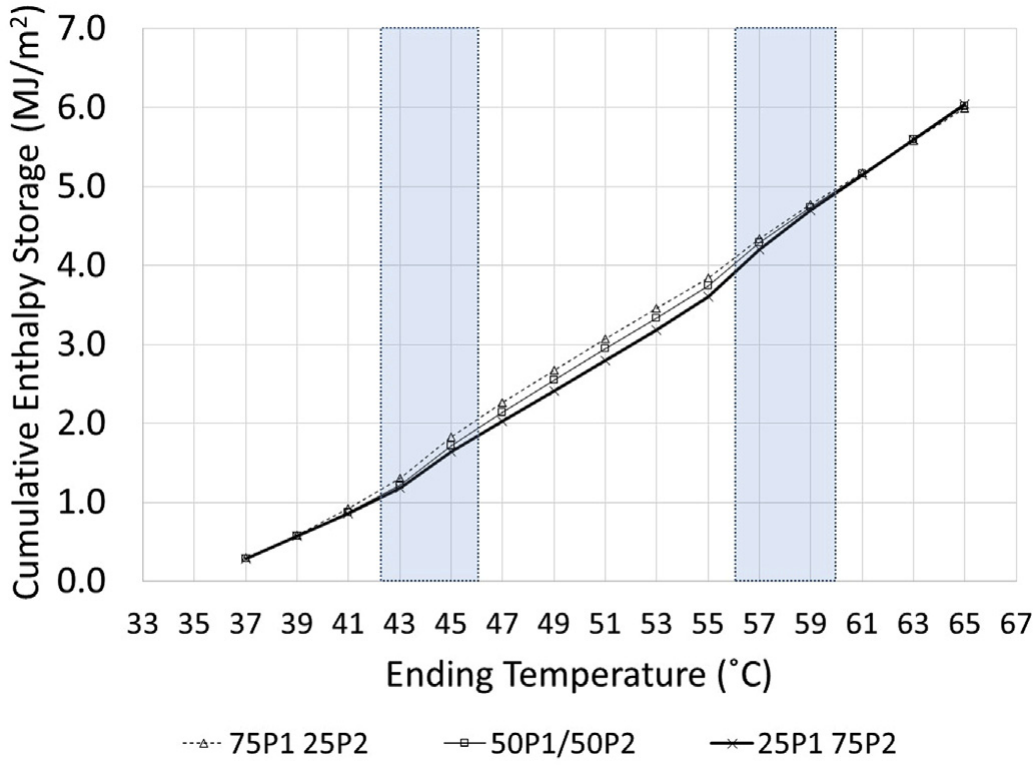
Influence of P1:P2 ratio and temperature on Cumulative Enthalpy of Concrete Containing Hybrid PCM.

In this case of concrete containing PCM in hybrid form, three specific heats can be determined based on the state of each PCM: C_pS_ is the specific heat where both PCMs are in solid state, C_pL/S_ is the specific heat where one PCM is in liquid state and another is still in solid state, and C_pL_ is the specific heat where both PCMs are in liquid state. The relationship between cumulative enthalpy and temperature is shown in Figure 10 and Table 8.

**Table 8:** Latent heat.

Concrete type	Heat (MJ/m^2^)	Temperature range (^o^C)		Summation (MJ/m^2^)
		43–47	55–59	
100P_1_/0P_2_	Total heat	1.44	-	1.44
	Sensible heat	0.89	-	0.89
	Latent heat	0.55	-	0.55
75P_1_/25P_2_	Total heat	1.35	1.33	2.68
	Sensible heat	0.87	1.01	1.88
	Latent heat	0.48	0.32	0.80
50P_1_/50P_2_	Total heat	1.26	1.42	2.68
	Sensible heat	0.85	1.01	1.86
	Latent heat	0.42	0.40	0.82
50P_1_/50P_2_	Total heat	1.18	1.49	2.67
	Sensible heat	0.83	1.00	1.84
	Latent heat	0.34	0.49	0.83
0P_1_/100P_2_	Total heat	0	1.56	1.56
	Sensible heat	0	1.02	1.02
	Latent heat	0	0.54	0.54

The specific heat of concrete with hybrid PCMs depended strongly the PCM state and the PEG/PRF ratio in each concrete type. The value of C_pL/S_ fell intermediately between C_pS_ and C_pL_ regardless of the concrete type. In this specific case of C_pL/S_, the concrete was tested under the condition of PEG in a liquid state and PRF in a solid state. The phase of PEG in aggregates that underwent change from solid to liquid state caused the C_pL/S_ to increase to be slightly higher than C_pS_. As the PEG aggregate content decreased, the value of C_pL/S_ also decreased. This can be seen by the decreasing of Cp_L/S_ from 0.198 to 0.187 MJ/m^2^/^o^C with the decrease of PEG/PRF ratio from 75/25 to 25/75. As the temperature continuously increased, the concrete then approached a temperature high enough to cause both PCMs to change phase to liquid state, this was a region where the C_p_ became the highest and C_pL_ was measured.

#### 3.5.3 Latent heat of concrete

**Figure 11:**
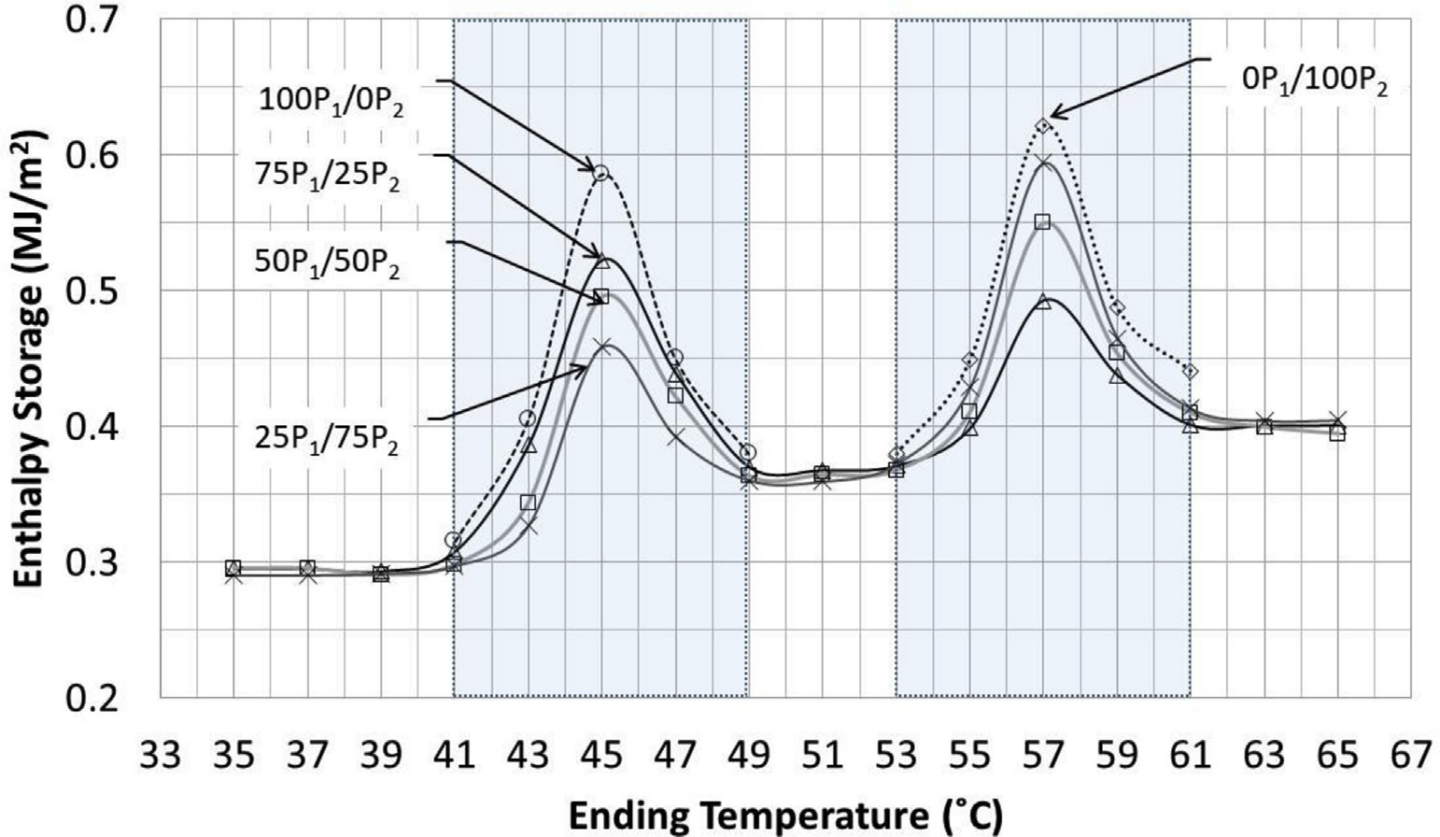
Relationship between Enthalpy Storage vs. Temperature.

The latent heat can be measured from the enthalpy storage over ranges of PCM active temperature using Eqs. (2), (3), (4), (5), (6), and (7). The results are shown in Table 8 and Figure 11 in form of the relation between enthalpy storage and temperature. In term of heat profile, the graph can be divided into 5 regions based on temperature ranges.

• The first region was when both PCMs were in solid state and the temperature was in the range of 35–41 °C. Since the PCMs were still in a solid state, there was no effect of latent heat. The heat storage in this zone was a sensible heat of concrete with both PCMs in solid state.
• The second region occurred in the melting temperature range of PEG (41–49 °C). In this region, the PEG began to melt and stored heat in form of latent heat. The latent heat storage for each concrete type depended strongly on the ratio of PEG aggregates.
• After all the PEG melted entirely, the concrete shifted to the third region where latent heat had no influence the heat storage. The heat stored in this region was considered the sensible heat storage of concrete with one melted PCM and one solid PCM.
• As the temperature continued to increase to the melting range of PRF (55–61 °C), the PRF began to melt and stored latent heat. The amount of latent heat storage depended on the ratio of PRF aggregates in each concrete type.
• The last region occurred when both PCMs were in liquid state (temperature higher than 61 °C). The heat storage observed in this region was the sensible heat of concrete with both PCMs in aggregates in liquid state.

Table 8 shows the results in term of values of total, sensible and latent heats. Both 100P_1_/0P_2_ and 0P_1_/100P_2_ concretes which contained single type PCM aggregates were subjected to on region of heat with respected to the melting point of their PCM (either 43–47 or 55–59 °C, for PEG and PRF type). The latent heat of 0.54–0.55 MJ/m^2^ was observed. For concrete containing hybrid PCM aggregates, higher latent heat of 0.80–0.83 MJ/m^2^ was observed. This is because both type of concretes were subjected to heat in two regions (both 43–47 or 55–59 °C). This indicated that the use of concrete with PCM in hybrid form provide higher latent heat and become more effective in storing heat than those with single type PCM.

## 4 Conclusions

• The use of PCM aggregates enhanced the properties of concrete in several aspects. These included the effect of PCM filling voids in aggregates caused the aggregates to become denser and less absorptive which led an improvement in workability, an increase in density, and a decrease in moisture absorption. The compressive strength and abrasion resistance also increased when conventional aggregates were replaced with PCM aggregates.
• The thermal conductivity (k) of PCM lightweight concrete was found to depend strongly on the state of PCM. The k_25_ (k of PCM in solid state) increased with the PCM aggregate content due to the reduction in void content which was replaced by the solid PCM. The k_65_ (k of PCM in liquid state), on the other hand, decreased with the PCM aggregate content due to the effect of latent heat during phase changing process. The k_25_ and k_65_ were found in the range of0.829–0.842 and 0.447–0.465 W/m^°^C, respectively.
• For both types of concrete (single and hybrid PCM aggregate), the specific heat was found to increase as the state of PCM. The concretes with PCM in liquid form yielded higher specific than those with PCM in solid form.
• For the PCM aggregate in hybrid form, at the intermediate temperature region where the concrete containing both solid and liquid PCM, the specific depended strongly on the quantity of PCM in liquid form. The concretes with higher content of liquid PCM aggregates yielded higher specific heat that those with lower content of liquid PCM.
• The latent heat of concrete with PCM aggregates in hybrid form were found to be higher than those with single type PCM aggregates. This indicated that concrete with PCM in hybrid form can be more effective in storing heat at high temperature than concrete with singly type PCM aggregates.

## Declarations

### Author contribution statement

Piti Sukontasukkul: Conceived and designed the experiments; Wrote the paper.

Teerawat Sangpet: Performed the experiments.

Moray Newlands & Doo-Yeol Yoo: Analyzed and interpreted the data; Wrote the paper.

Weerachart Tangchirapat: Analyzed and interpreted the data.

Suchart Limkatanyu & Prinya Chindaprasirt: Contributed reagents, materials, analysis tools or data.

### Funding statement

This work was supported by King Mongkut’s University of Technology North Bangkok under contract no. KMUTNB-63-KNOW-024, the 10.13039/501100004396Thailand Research Fund under contract no. RTA6280012 (Suchart Limkatanyu) and DPG6180002 (Prinya Chindaprasirt).

### Competing interest statement

The authors declare no conflict of interest.

### Additional information

No additional information is available for this paper.
